# Cognitive Behavioral Therapy as a Game Changer in HIV Prevention for Vulnerable Populations: A Systematic Review and Meta-Analysis

**DOI:** 10.7759/cureus.77560

**Published:** 2025-01-16

**Authors:** Hosam Hadi Hassan Awaji, Wadeea H Awaji, Foad M Tohari, Abdulitef A Alamrani, Ibrahim Y Hamdi, Turki Y Aljuhani, Ahmad M Alatawi, Mohammed E Alatawi, Faisal A Alhowiti, Bandar M Albalawi, Mohammed I Al-Asiri

**Affiliations:** 1 Preventive Medicine Department, King Salman Armed Forces Hospital, Tabuk, SAU; 2 Nursing-Preventive Medicine Department, North West Armed Forces Hospital, Tabuk, SAU; 3 Laboratory Department, North West Armed Forces Hospital, Tabuk, SAU; 4 Public Health Department, North West Armed Forces Hospital, Tabuk, SAU; 5 Nursing Department, North West Armed Forces Hospital, Tabuk, SAU

**Keywords:** cbt, cognitive behavioral therapy, high-risk population, hiv prevention, meta-analysis

## Abstract

Cognitive behavioral therapy (CBT) is a promising intervention for HIV prevention among high-risk individuals. However, its efficacy compared to standard counseling remains unclear. This meta-analysis aimed to evaluate the effectiveness of CBT in reducing HIV risk behaviors. A systematic review and meta-analysis were conducted to identify randomized controlled trials comparing CBT to standard counseling for HIV prevention from inception to November 30, 2024. Primary outcomes included sexual transmission risk behavior and the number of unprotected anal intercourse (UAI). Secondary outcomes included alcohol use, substance use, and suicidality. Nine studies with a total of 3189 participants were included in the meta-analysis. CBT did not significantly reduce sexual transmission risk behavior compared to standard counseling. However, CBT was associated with a significant reduction in UAI at the six- and 12-month post-intervention. Regarding secondary outcomes, CBT led to a significant reduction in substance use, while standard counseling was more effective in reducing alcohol use. CBT appears to be effective in reducing specific high-risk behaviors, particularly UAI, among high-risk individuals. However, the mechanisms of action of CBT and its long-term effects require further investigation.

## Introduction and background

Cognitive behavioral therapy (CBT) has evolved significantly since its inception in the mid-20th century. Initially developed by Aaron Beck and Albert Ellis, CBT emerged as a fusion of behavioral and cognitive therapies aimed at altering dysfunctional thinking patterns and behaviors. Over the decades, CBT has diversified into various methods, including Rational Emotive Behavior Therapy (REBT), Dialectical Behavior Therapy (DBT), and Mindfulness-Based Cognitive Therapy (MBCT), each tailored to address specific psychological issues [1].

CBT's application in HIV prevention is particularly noteworthy. It focuses on modifying high-risk behaviors and enhancing coping mechanisms, which are crucial for individuals at risk of HIV. CBT interventions often include stress management, problem-solving skills, and behavioral strategies to reduce risky sexual behaviors and improve medication adherence. These interventions have shown efficacy in reducing HIV transmission rates and improving the quality of life for those living with HIV [2].

Standard counseling practices in HIV prevention emphasize personalized risk assessments, education on safe practices, and continuous support. These practices are integral to CBT, which aims to empower individuals with the knowledge and skills to manage their health proactively [3].

High-risk groups for HIV include men who have sex with men (MSM), people who inject drugs (PWID), sex workers, and transgender individuals. These populations often face significant barriers to accessing healthcare and are disproportionately affected by HIV. CBT's structured approach can help mitigate these barriers by providing tailored interventions that address the unique challenges faced by these groups [4].

The aim of this meta-analysis is to systematically evaluate the effectiveness of CBT in preventing HIV among high-risk groups. By synthesizing data from various studies, this analysis seeks to determine the overall impact of CBT on reducing high-risk behaviors, improving adherence to preventive measures, and enhancing the psychological well-being of individuals at risk of HIV. Additionally, the meta-analysis aims to compare the efficacy of CBT with standard counseling practices, providing a comprehensive understanding of its role in HIV prevention.

## Review

Methods

This meta-analysis was conducted and reported in accordance with the principles of the Cochrane Handbook for Systematic Reviews of Interventions, version 6, and the Preferred Reporting Items for Systematic Reviews and Meta-Analyses (PRISMA) guidelines [5].

Aim of the study

The purpose of this study was to compare the effectiveness of CBT with standard counseling practices in preventing HIV, with the goal of quantifying reductions in high-risk behaviors (e.g., unprotected sex and needle sharing) among individuals receiving CBT interventions.

Eligibility criteria for the included studies

This meta-analysis included randomized controlled studies that were published from inception to November 30, 2024. Eligible studies included all that compared graduated and progressive compression in chronic venous disorders. No restrictions were implemented regarding the age, sex, or race of the participants. Studies that were considered eligible included those that compared CBT with standard counseling practices or other interventions aimed at HIV prevention in high-risk groups. We excluded studies involving animals, retrospective analyses, conference abstracts, duplicate entries, case reports, review articles, commentaries, case series with fewer than four patients, or clinical guidelines.

Search strategy and selection of studies

The following electronic databases were searched for eligible studies: MEDLINE/PubMed, Cochrane Central Register of Controlled Trials (CENTRAL), Web of Science, ProQuest, and Scopus. The search was set for all articles published in English from inception until November 30, 2024. The following search terms were used: (cognitive behavioral therapy) AND (HIV counseling and testing OR psychological counseling) AND (HIV behavioral risk). We used no filters by language or publication period. The first reviewer searched within the reference lists of articles that had been obtained for other potentially relevant studies that had not been retrieved by electronic search. The first reviewer screened the retrieved reports for eligibility through title, abstract, and full-text screening. The second reviewer checked the retrieved studies, and discrepancies were solved through discussion with a third reviewer.

Data extraction

The first reviewer carried out data extraction from the included studies using a standardized data sheet, which included (a) the study's characteristics (author, year, country, study design); (b) patient characteristics (age at the time of treatment, sex, sample size); (c) intervention details (type and the duration of follow-up); and (d) outcomes (primary outcomes: total number of unprotected anal intercourse (UAI) events and HIV behavioral risk; secondary outcomes: alcohol use and substance use). The second reviewer checked the collected data for consistency and clarity. Any disagreements were settled by consulting the third reviewer.

Measured outcomes

The primary outcomes included a reduction of sexual transmission risk behavior among high-risk individuals at four and eight months and a reduction of the total number of UAI among high-risk individuals at three, six, and nine months. Secondary outcomes included alcohol use (Alcohol Use Disorders Identification Test, AUDIT) score reduction after treatment, substance use (Short Inventory of Problems-Alcohol and Drugs, SIP-AD) score reduction after treatment, and suicidality (Suicidal Ideation Attributes Scale, SIDAS) score reduction after treatment.

Assessment of the risk of bias in the included studies

The risk of bias in the included studies was assessed using the National Institute for Health and Care Excellence (NICE) checklists for randomized controlled clinical trials [6].

Data synthesis

**Figure 1:**
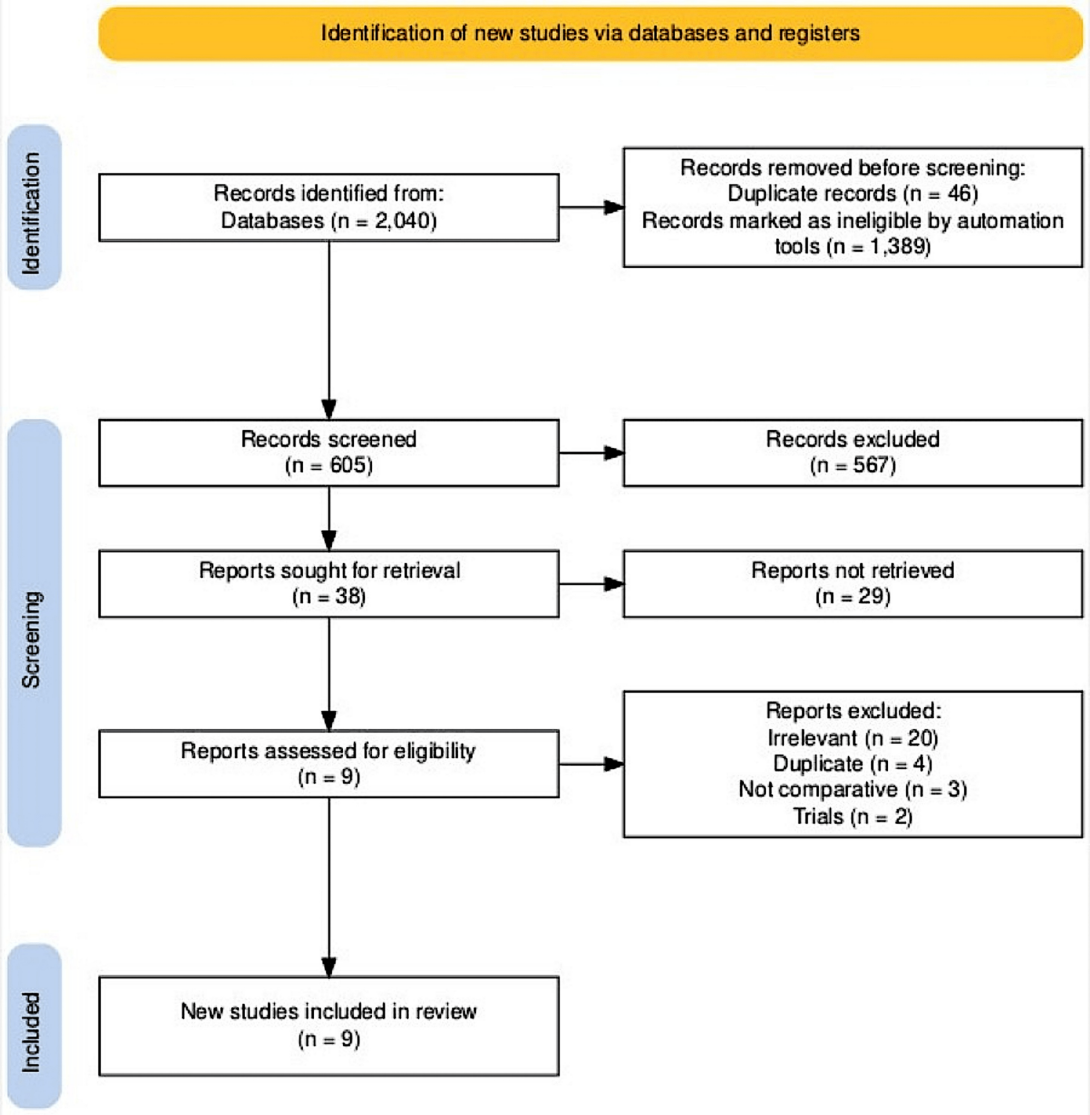
PRISMA flowchart PRISMA: Preferred Reporting Items for Systematic Reviews and Meta-Analyses

Initially, 2040 records were retrieved from electronic database searches. Following the elimination of duplicates and excluded studies, 38 studies were deemed eligible. Among these, nine studies [7-15] involving 3189 individuals were included (Table 1). The 29 studies excluded from the meta-analysis were either irrelevant (n=20), duplicates (n=4), non-comparative (n=3), or trials (n=2), as illustrated in Figure 1 [16].

**Table 1:** Summary of the included studies NM: not mentioned; RCT: randomized controlled trials; CBT: cognitive behavioral therapy; MSM: men who have sex with men; TW: transgender women

Author	Year	Country	Study Design	Age		Sex (M: F)		Sample Size		Primary Outcomes	Secondary Outcomes	Follow-Up Duration
				CBT	standard	CBT	standard	CBT	standard			
Coffin et al. [7]	2014	USA	RCT	Mean (SD): 34 (10.5)	33.2 (9.6)	Men	Men	162 (completed 3=150, 6=151)	164 (completed 3=157, 6=161)	Total number of UAI (unprotected anal intercourse) events, number of UAI partners, number of UAI events with three most recent non-primary partners	Number of SDUAI (Substance-Dependent Unprotected Anal Intercourse) events, condom-protected anal intercourse events, number of insertive UAI events	Baseline, 3 months, 6 months
Hershberger et al. [8]	2003	USA	RCT	38.43+38.57 +39.04	38.43+38.57 +39.04	F (33.01+ 33.33+ 32.81)	F (33.01+ 33.33+ 32.81)	281	487	Drug use and HIV risk behaviors	NM	Between 5 and 9 months after baseline
Dilley et al. [9]	2002	USA	RCT, four-arm	32.7	32.6	Men	Men	124 (62 with diary, 62 without)	124 (62 with diary, 62 without)	Mean change of episodes of UAI	NM	Baseline, 6 months, 12 months
Dilley et al. [10]	2011	USA	RCT	28 (4.95)	28 (4.95)	Men	Men	147	158	Reduction in unprotected anal intercourse (UAI) episodes (high-risk sex)	Satisfaction with services received (quality of service, counselor competence)	Baseline, 6 months, and 12 months
Yi et al. [11]	2024	China	RCT	23.73 (3.36)	22.72 (3.09)	Men	Men	60 (completed 54)	60	HIV-Transmission-Risk Behavior Measured as condomless anal sex acts	HIV Social-Cognitive Mechanisms, Mental and Behavioral Health Outcomes, Minority Stress Mechanisms, Universal Mechanisms	Baseline, 4 months, and 8 months
Mansergh et al. [12]	2010	USA	RCT (2 randomized, control is nonrandomized)	37.02 (6.75)	37.31 (6.75)	Men	Men	599	480	Reduction in unprotected anal intercourse (UAI) and HIV-discordant UAI (DUAI)	Reduction in unprotected receptive/insertion sex, substance use before/during UAI	3, 6, and 12 months
O’Cleirigh et al. [13]	2019	USA	RCT	38.87 (11.68)	39.55 (10.61)	Men	Men	23	20	Reduction in condomless sex acts (especially with Sero discordant partners), reduction in posttraumatic symptom severity (Davidson Trauma Scale)	Changes in Davidson Trauma Scale subscales: Avoidance, Intrusions, Hyperarousal Maintenance effects at 3-, 6-, and 9-month follow-up Individual variation in treatment response	Baseline, 3, 6, and 9 months
Pachankis et al. [14]	2022	USA	RCT, three-arm	Mean age: 26.55 years (SD = 4.17)	Mean age: 26.55 years (SD = 4.17)	Predominantly male (98.1% cisgender)	Predominantly male (98.1% cisgender)	101	52 (the third arm included 100 undergoing ESTEEM, which was not included in the study)	Reduction in HIV-transmission-risk behaviors, reduction in mental health comorbidities (MINI-derived diagnoses)	Mental health outcomes: depression, anxiety, suicidality, psychological distress. Behavioral health outcomes: substance use, transdiagnostic outcomes: number of comorbidities from baseline to follow-up	8 months (main analysis), 12 months (additional follow-up)
Passaro et al. [15]	2020	USA	RCT	24.5 (21, 29)	24 (21, 30)	118 MSM, 31 TW	118 MSM, 31 TW	50	103	Reduction in prevalence of condomless receptive anal intercourse (cRAI)	Changes in AUDIT and DEQ-MSM scores, incidence of HIV, and rectal GC/CT infections	6 months (evaluations at 3 and 6 months)

Statistical analysis

Meta-analysis was performed using Review Manager (RevMan) 5.4 version (The Cochrane Collaboration, London, UK) [17]. Pooled mean differences (MD) were used for comparing various scores describing the sexual transmission behavior, number of UAI, alcohol use (AUDIT) score, substance use (SIP-AD) score, and suicidality (SIDAS) score among the pooled studies with 95% confidence intervals (CIs) calculated using a random-effects model due to expected heterogeneity among studies. Heterogeneity was assessed using the I^2^ statistic, with values above 50% indicating substantial heterogeneity. Sensitivity analyses were conducted by excluding studies one by one to determine the robustness of the results.

Results

Nine studies [7-15] were included in this meta-analysis with a total of 3189 participants to investigate the efficacy of CBT compared to standard counseling in HIV prevention among high-risk individuals.

Primary Outcomes

**Figure 2:**
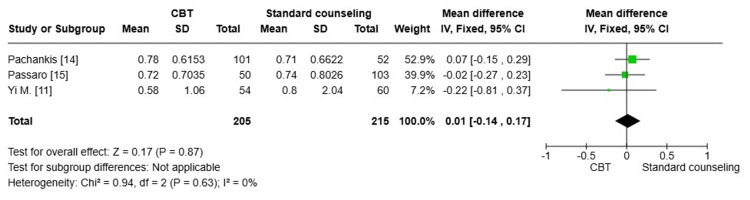
Forest plot comparing the sexual transmission risk behavior after four months of treatment CBT: cognitive behavioral therapy; SD: standard deviation

Sexual transmission risk behavior after four months of treatment: Three pooled studies [11,14,15] with a total number of 420 patients showed no statistically significant difference in sexual transmission risk behavior between the CBT group and the standard counseling group after four months of treatment (MD=0.01; 95% CI: -0.14, 0.17, p=0.87). The degree of heterogeneity among the studies was minimal, indicating a high level of consistency in the results (I^2^=0%) (Figure 2).

**Figure 3:**
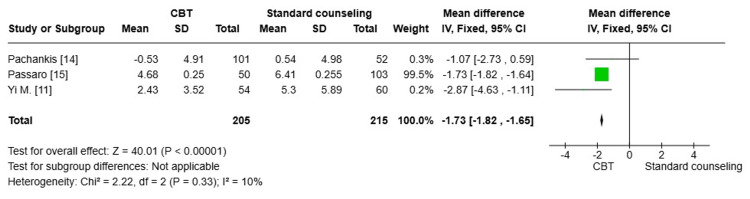
Forest plot comparing the sexual transmission risk behavior after eight months of treatment CBT: cognitive behavioral therapy; SD: standard deviation

Sexual transmission risk behavior after eight months of treatment: Two studies [11,14] (n=265) were pooled to compare the sexual transmission risk behavior scale between the CBT group and the standard counseling group. There was no statistically significant difference between both groups in reducing sexual transmission risk behavior among high-risk individuals after eight months of treatment (MD=0.04; 95% CI: -0.19, 0.26, p=0.75). The pooled studies showed minimal heterogeneity (I^2^=0%) (Figure 3).

**Figure 4:**
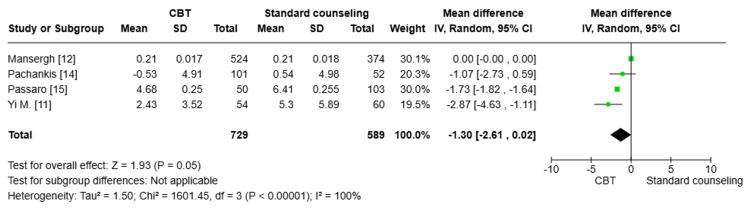
Forest plot comparing the number of unprotected anal intercourse (UAI) at three months CBT: cognitive behavioral therapy; SD: standard deviation

Number of UAI at three months: Four studies [7,12,13,15] were pooled (n=1394), and the result showed that there was no statistically significant difference in the reduction of UAI between the CBT group and the standard counseling group after three months of treatment (MD=0.27; 95% CI: -0.62, 1.16, p=0.55). There was substantial heterogeneity between the pooled studies (I^2^=99%); this heterogeneity was best resolved by the exclusion of Coffin et al. (Figure 4) [7].

**Figure 5:**
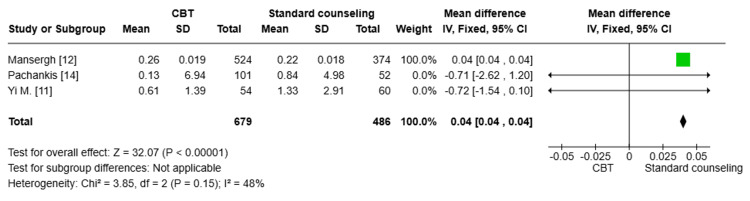
Forest plot comparing the reduction in unprotected anal intercourse (UAI) at six months CBT: cognitive behavioral therapy; SD: standard deviation

Number of UAI at six months: Five studies [7,9,10,13,15] with a total number of 1061 patients were pooled, and a statistically significant difference in the reduction of UAI between the CBT group and the standard counseling group after six months was found. The CBT group showed a greater reduction in UAI compared to the standard counseling group (MD= -0.18; 95% CI: -0.33, -0.03, p=0.02). There was significant heterogeneity among the pooled studies (I^2^=72%); this heterogeneity was best resolved by the exclusion of Diley J. W. et al. (Figure 5) [9].

**Figure 6:**
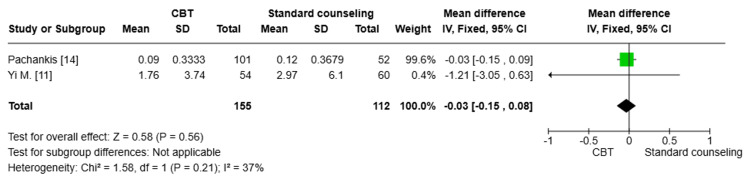
Forest plot comparing the reduction of unprotected anal intercourse (UAI) after 12 months CBT: cognitive behavioral therapy; SD: standard deviation

Number of UAI at 12 months: Two studies [9,10] (n=559) were pooled. There was a statistically significant difference in the reduction of UAI between the CBT group and the standard counseling group after 12 months (MD= -0.16; 95% CI: -0.17, -0.15, p<0.00001). The degree of heterogeneity among the studies was minimal, indicating a high level of consistency in the results (I^2^=0%) (Figure 6).

Secondary Outcomes

**Figure 7:**
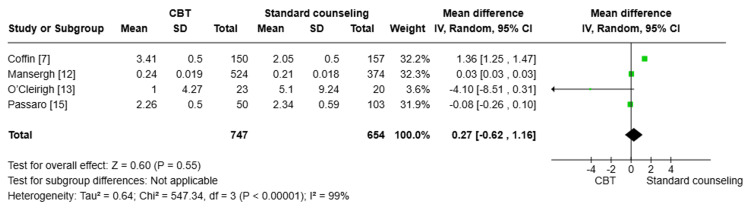
Forest plot comparing the reduction of alcohol use (AUDIT) between the CBT group and the standard counseling group CBT: cognitive behavioral therapy; SD: standard deviation

Alcohol use (AUDIT) score: Three studies [11,14,15] with a total number of 420 were pooled to compare the reduction of alcohol use (AUDIT) between the CBT group and the standard counseling group. There was a statistically significant reduction in alcohol use among the standard counseling group compared to the CBT group (MD= -1.73; 95% CI: -1.82, -1.65, p<0.00001). The degree of heterogeneity among the studies was minimal (I^2^=10%), indicating a high level of consistency in the results (Figure 7).

**Figure 8:**
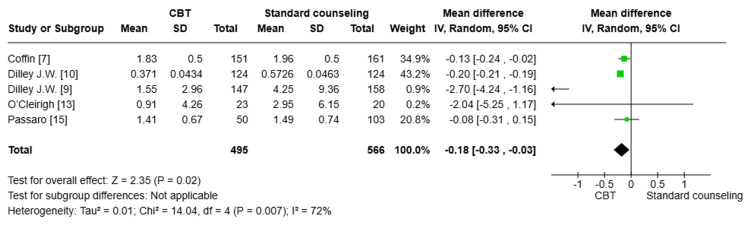
Forest plot comparing the substance use (SIP-AD) score between both groups CBT: cognitive behavioral therapy; SD: standard deviation

Substance use (SIP-AD) score: A pooled analysis of three studies [11,12,14] (n=1163) showed a statistically significant reduction in substance use (SIP-AD) in the CBT group compared to the standard counseling group (MD=0.04; 95% CI: 0.04, 0.04, p<0.00001). The degree of heterogeneity among the studies was moderate (I^2^=48%), indicating some level of inconsistency in the results (Figure 8).

**Figure 9:**
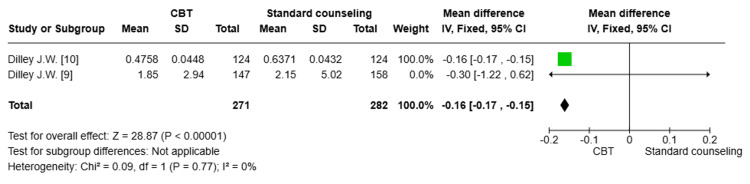
Forest plot comparing the reduction of suicidality (SIDAS) between the CBT group and the standard counseling group CBT: cognitive behavioral therapy; SD: standard deviation

Suicidality (SIDAS) score: Two studies [11,14] (n=267) were pooled to compare the reduction of suicidality (SIDAS) between the CBT group and the standard counseling group. There was no statistically significant difference between both groups (MD= -0.03; 95% CI: -0.15, 0.08, p=0.56). A moderate degree of heterogeneity was detected among the pooled studies (I^2^=37%) (Figure 9).

Risk of Bias

All studies had a low risk of bias regarding random sequence generation, indicating adequate randomization methods. Less than half of the studies (four out of nine) had a low risk of bias in the concealment of allocation to treatment groups. However, four studies (Hershberger et al., Dilley J.W. et al., O'Cleirigh et al., and Yi et al.) had an unclear risk of bias, indicating insufficient information about how allocation concealment was achieved [8,10,11,13]. Mansergh et al. did not mention allocation concealment, raising a high risk of bias in this domain [12].

Blinding of Participants and Personnel (Performance Bias)

**Figure 10:**
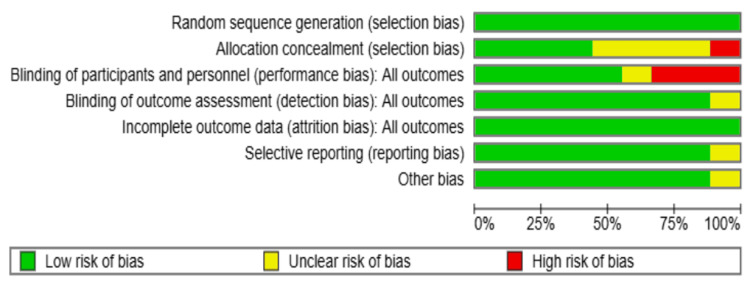
Risk of bias graph

**Figure 11:**
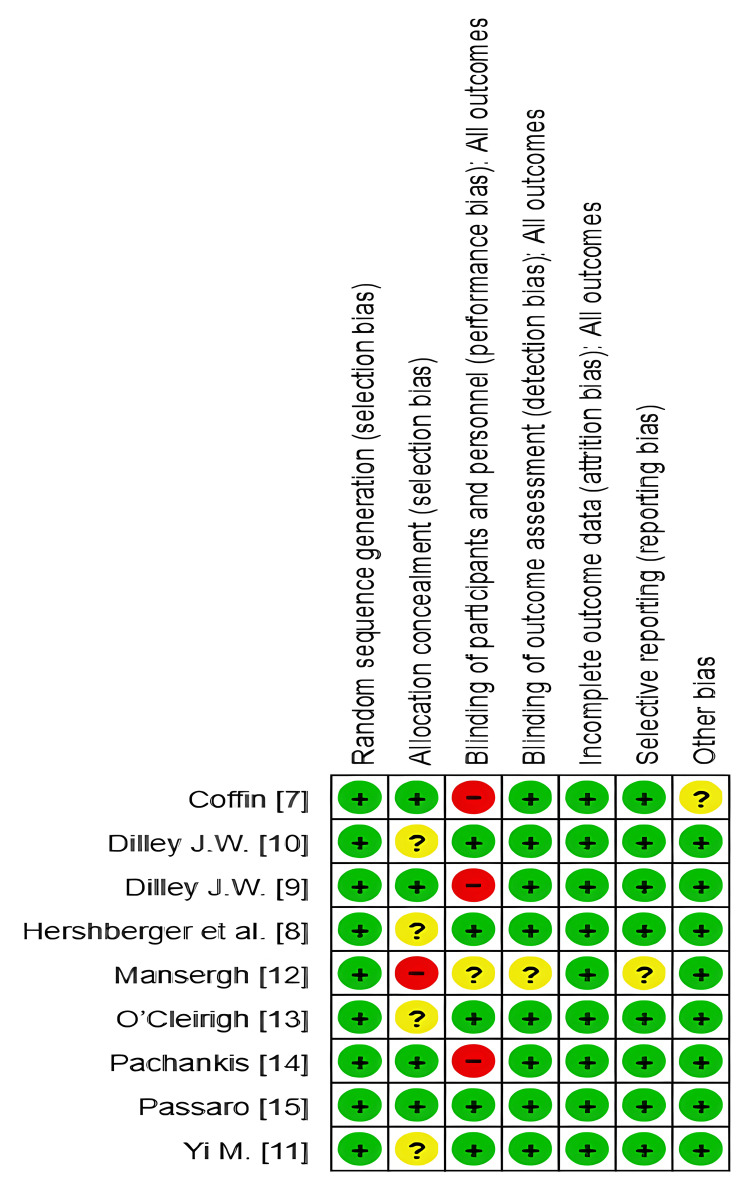
Risk of bias summary

Three studies [7,9,14] exhibited a high risk of performance bias, whereas one study [12] did not mention adequate data regarding participant blinding. This could be attributed to the fact that it is unlikely to achieve adequate blinding in this type of intervention. Blinding of outcome assessment (detection bias) was adequately made in most studies (eight out of nine) [7-15]. However, one study [12] had an unclear risk of bias, suggesting insufficient information about how blinding was achieved. All studies showed a low risk of attrition bias, indicating adequate handling of missing data. However, reporting bias was detected in one study, whereas the rest of the studies had adequate reporting of outcomes, except for Coffin et al. [7], which showed a potential source of bias; all other studies had no other risk of bias (Figures 10, 11).

Discussion

CBT is a form of psychotherapy that helps individuals identify and change negative thought patterns and behaviors. It has been widely used in various psychological interventions, including HIV prevention. Standard counseling, on the other hand, is a more traditional approach to therapy that often involves providing support, advice, and education [2]. HIV prevention among high-risk groups is a critical public health issue. High-risk groups, such as MSM, transgender individuals, and PWID, are disproportionately affected by HIV infection. To address this issue, various interventions have been developed, including CBT and standard counseling [18].

This meta-analysis aimed to evaluate the efficacy of CBT compared to standard counseling in HIV prevention among high-risk individuals. While the results are promising, they also present a complex picture. Regarding primary outcomes, the analysis demonstrated that CBT did not significantly reduce sexual transmission risk behavior after four or eight months of treatment. However, a significant reduction in the number of UAI was observed at the six- and 12-month post-treatment. This suggests that while CBT may not immediately impact overall sexual risk behavior, it can effectively reduce specific high-risk behaviors over time.

In terms of secondary outcomes, the analysis revealed a significant reduction in substance use among CBT participants compared to the standard counseling group. Conversely, the standard counseling group showed a significant reduction in alcohol use. These findings highlight the potential differential impact of the two interventions on specific behaviors.

Limitations

Several limitations should be considered when interpreting the results of this meta-analysis. One significant limitation is the heterogeneity observed in some pooled analyses, particularly for the number of UAI at three and six months. This suggests that the underlying mechanisms of action of CBT may vary across studies, potentially due to differences in participant characteristics, intervention intensity, or cultural factors.

Another limitation is the risk of bias in some studies, particularly regarding allocation concealment and blinding of participants and personnel. While most studies had a low risk of bias, these limitations may have influenced the results and should be considered when interpreting the findings. Additionally, the generalizability of the findings may be limited to MSM and transgender individuals, as the included studies primarily focused on these populations. Further research is needed to assess the effectiveness of CBT in other high-risk groups, such as heterosexual individuals or PWID.

Furthermore, the included studies had relatively short follow-up periods. Long-term studies are needed to assess the sustained efficacy of CBT in HIV prevention. Finally, while the meta-analysis provides evidence for the effectiveness of CBT, it does not fully elucidate the underlying mechanisms through which CBT exerts its effects. Future research should investigate the specific cognitive and behavioral processes contributing to the observed outcomes.

## Conclusions

The meta-analysis evaluated the efficacy of CBT compared to standard counseling in HIV prevention among high-risk individuals. Across various outcomes, CBT demonstrated varying levels of effectiveness. For sexual transmission risk behavior, neither the four-month nor the eight-month follow-up period showed any significant differences between the two interventions. Regarding UAI, standard counseling was significantly more effective at six and 12 months, but results were mixed at three months. Standard counseling was more significant in reducing alcohol use (AUDIT); on the other hand, CBT was statistically significant in reducing substance use (SIP-AD) compared to standard counseling. However, there was no significant difference in the reduction of suicidality (SIDAS) between the two interventions. While these findings suggest potential benefits of standard counseling, the substantial heterogeneity observed in some analyses underscores the need for further research to identify optimal interventions and target populations.
